# Androgen deprivation therapy is associated with decreased second primary lung cancer risk in the United States veterans with prostate cancer

**DOI:** 10.4178/epih.e2018040

**Published:** 2018-08-11

**Authors:** Kyungsuk Jung, Jong Chul Park, Hyunseok Kang, Johann Christoph Brandes

**Affiliations:** 1 Department of Medicine, Fox Chase Cancer Center, Philadelphia, PA, USA; 2 Department of Medicine, Massachusetts General Hospital/Harvard Medical School, Boston, MA, USA; 3 Department of Oncology, Johns Hopkins University School of Medicine, Baltimore, MD, USA; 4 Department of Oncology, Atlanta Veterans Affairs Medical Center, Atlanta, GA, USA; 5 Department of Hematology and Medical Oncology, Emory University School of Medicine, Atlanta, GA, USA

**Keywords:** Lung neoplasms, Prostatic neoplasms, Androgen antagonists, Hormones, Veterans

## Abstract

**OBJECTIVES:**

We investigated whether androgen deprivation therapy (ADT) in prostate cancer patients was associated with a decreased risk for second primary lung cancer in US veterans.

**METHODS:**

Prostate cancer diagnoses in the US Veterans Affairs Cancer Registry between 1999 and 2008 were identified. Use of hormonal therapy and diagnoses of second primary lung cancer were determined from the registry. Synchronous prostate and lung cancers, defined as 2 diagnoses made within 1 year, were excluded from the analysis. Cancer-free survival was estimated using the Kaplan-Meier method and hazard ratios were estimated using Cox proportional hazard models.

**RESULTS:**

Among the 63,141 identified patients with prostate cancer, 18,707 subjects were eligible for the study. Hormonal therapy was used in 38% of patients and the median follow-up period was 28 months. ADT use was associated with longer lung cancer-free survival in prostate cancer patients (log-rank p=0.01). After adjusting for age, race, smoking and prostate cancer stage, ADT use was associated with decreased lung cancer risk by 15, 21, and 24% after 1, 2, and 3 years, respectively.

**CONCLUSIONS:**

ADT in prostate cancer patients may be associated with decreased second primary lung cancer risk among US veterans.

## INTRODUCTION

The endocrine system has been implicated in the development and treatment of various cancers, including breast, prostate, uterine, testicular, ovarian, and thyroid cancers [1]. Recently, increasing evidence has implicated estrogen in the development and prognosis of lung cancer. Even after adjusting for smoking history and body size, women have been shown to be at a higher risk for developing lung cancer in a few studies [2,3]. In a post hoc analysis of the Women’s Health Initiative study, use of a hormone supplement with conjugated equine estrogen and medroxyprogesterone acetate was shown to be significantly associated with increased mortality from non-small cell lung cancer (NSCLC) [4], while estrogen blockade with tamoxifen in breast cancer patients was shown to be associated with decreased mortality from lung cancer [5]. In addition to estrogen, androgens may also play a distinct role in lung cancer development. Androgen receptors (ARs) are expressed in normal lung tissues [6], as well as in cancer cells, including NSCLC [7,8]. The tumor cell proliferation in NSCLC induced by tobacco carcinogens (4-methylnitrosoamino-1-3-pyridyl-1-butanone and benzo[a]pyrene) was shown to be suppressed in AR-knockdown mice [9].

Androgen deprivation therapy (ADT) with a luteinizing hormone-releasing hormone (LHRH) agonist has been widely adopted in prostate cancer treatment. It suppresses the biosynthesis of both androgens and estrogens, because estrogens in men are mostly derived from the aromatization of testosterone [10]. Several observations have raised the possibility that suppression of sex hormones through ADT may decrease the risk of lung cancer. Analyses of the Surveillance, Epidemiology, and End Results (SEER) database showed that survivors of prostate cancer, who had possibly been treated with ADT, were at a lower risk of developing subsequent lung cancer than the general US population [11,12]. In another study, men lung cancer patients who had prior orchiectomy were shown to have significantly longer survival [13]. Based on these findings, we hypothesized that the use of ADT in prostate cancer patients may lead to a decreased risk of second primary lung cancer.

## MATERIALS AND METHODS

### Data source

Diagnoses of prostate cancer in US veterans seen in the Veterans Affairs (VA) system between January 1, 1999 and December 31, 2008 were identified from the Department of Veterans’ Affairs Central Cancer Registry (VACCR). The VACCR has been reported to capture at least 90% of patients with cancer who are treated in the VA system [14]. The primary outcome was a new diagnosis of lung cancer in the VACCR, which was followed until December 31, 2010. Stage and histopathology information, use of hormonal treatment, age at prostate cancer diagnosis, and race were also retrieved from the VACCR. Alcohol use and smoking history were obtained from the National Veterans Affairs Medical SAS dataset and were merged with data obtained from the VACCR using scrambled social security numbers as unique identifiers. Participants were categorized by smoking history as smokers, former smokers, and never smokers. This study was approved by the institutional review board of Emory University and the research and development committee of the Atlanta VA Medical Center.

### Study population

From the initial cohort of patients with a new diagnosis of prostate cancer between 1999 and 2008, we excluded patients with lung cancer diagnosed within 1 year after the diagnosis of prostate cancer to eliminate cases with synchronous cancers. Patients with an unknown smoking history were also excluded, as cigarette smoking is a widely known risk factor for lung cancer [15,16]. We also excluded patients without staging information for prostate cancer, as staging may serve as a major confounder in the determination of overall survival.

**Figure 1.**
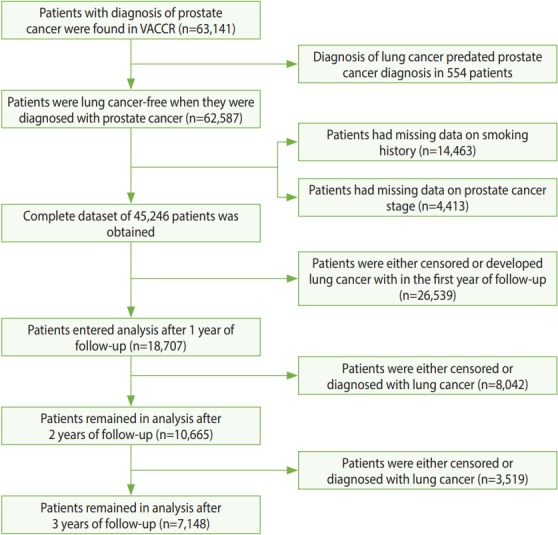
Identification and processin of patients’ data. VACCR, Veterans’ Affairs Central Cancer Registry.

Among the 63,141 patients with prostate cancer identified in the VACCR between January 1, 1999 and December 31, 2008, 554 were excluded because their lung cancer diagnoses preceded prostate cancer diagnoses. Fourteen thousand four hundred and sixty three did not have documentation of their smoking history, and 4,413 patients did not have information about their prostate cancer stage. Furthermore, 26,539 patients were either censored or developed lung cancer within the first year of follow-up, and thus were eliminated. Among the 18,707 patients in the final cohort, 7,148 were found to have received ADT (Figure 1).

The use of ADT was determined based on the information regarding the use of hormone therapy in the VACCR. In the Facility Oncology Registry Data Standards, hormonal therapy is defined as ‘Cancer therapy that achieves its antitumor effect through changes in hormonal balance. This includes the administration of hormones, agents acting via hormonal mechanisms, antihormones and steroids’ [17]. As the sojourn time for second primary lung cancer in patients receiving ADT is not known, we analyzed lung cancer-free survival for patients followed for 1, 2, and 3 years from the diagnosis of prostate cancer.

### Statistical analysis

All statistical analyses were carried out using 2-sided tests with the statistical significance level set at a p-value of 0.05. The basic characteristics of patients who received ADT and patients who did not receive ADT were compared using the chi-square test or the Student *t*-test. Lung cancer-free survival rates were evaluated and compared between the 2 groups using Kaplan-Meier curves and the log-rank test. Multivariable Cox proportional hazards models were used to estimate the hazard ratios (HRs) and 95% confidence intervals (CIs) reflecting the association between ADT and lung cancer incidence. The analysis was adjusted for age, race, smoking status, and prostate cancer stage. All statistical analyses were carried out using SAS version 9.2 (SAS Institute Inc., Cary, NC, USA) or Stata version 10 (StataCorp., College Station, TX, USA).

## RESULTS

### Basic characteristics of the study population

In the final cohort of 18,707 patients, the median age was higher in the ADT group than in the non-ADT group, which reflects pre-existing patterns of practice [18]. The racial composition between the ADT group and non-ADT group was found to be significantly different based on analysis of variance, but the overall distribution was comparable. There were more smokers in the ADT group, but overall the numbers of former smokers, current smokers, and never smokers seemed to be balanced. More patients with a higher stage of prostate cancer at the time of diagnosis received ADT, which reflects current treatment guidelines. The follow-up periods were comparable between the 2 groups (mean follow-up: 37.0 vs. 36.4 months; p=0.883) (Table 1).

The basic characteristics of patients who were followed for at least 2 or 3 years were similar to the patient cohort who were followed for at least 1 year. Among the patients with at least 2 or 3 years of follow-up, the mean follow-up period was comparable in the ADT and non-ADT groups, although there were significant differences in mean age and prostate cancer stage in the patients with more than 1 year of follow-up (Supplementary Material 1).

### Risk of second primary lung cancer

Among the 18,707 patients in the studied cohort, 572 subsequently developed second primary lung cancer. Patients who received ADT were less likely to develop lung cancer than those who did not receive ADT (log-rank p=0.01). When adjusted for age, smoking status, prostate cancer stage, and race, ADT use was associated with a non-significant reduction of lung cancer risk by 15% (HR, 0.85; 95% CI, 0.71 to 1.12; p=0.073) (Table 2). In patients with longer follow-up periods, ADT use was associated with a significant reduction of lung cancer risk by 21% at 2 years (HR, 0.79; 95% CI, 0.64 to 0.97) and by 24% at 3 years (HR, 0.76; 95% CI, 0.59 to 0.97) (Table 2).

### Racial differences in the effect of ADT on different types of cancer

**Figure 2.**
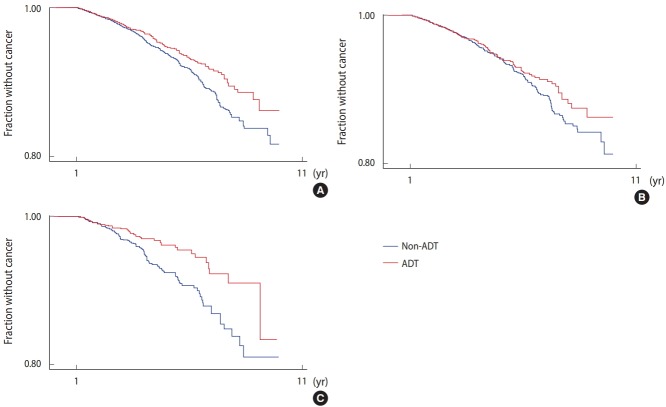
Kaplan-Meyer curve of lung cancer free survival in different races. (A) Lung cancer free survival in all races. (B) Lung cancer free survival in Caucasians. (C) Lung cancer free survival in African-Americans.

In subgroup analyses divided by race (Caucasian vs. African-American), ADT use seemed to be significantly associated with a reduction of lung cancer risk in African-Americans (HR, 0.56; 95% CI, 0.38 to 0.82; p=0.003) (Figure 2 and Table 3). In Caucasians, ADT use was not significantly associated with reduced lung cancer risk (HR, 0.97; 95% CI, 0.79 to 1.19; p=0.743) (Table 3). The effect of ADT on lung cancer risk reduction in African-Americans was largely limited to NSCLC (HR, 0.61; 95% CI, 0.41 to 0.89; p=0.011), while no such association was observed for small cell lung cancer (SCLC) in either Caucasians (HR, 0.98; 95% CI, 0.57 to 1.68) or African-Americans (HR, 0.13; 95% CI, 0.01 to 1.11; p=0.062) (Table 3).

## DISCUSSION

Our analysis suggests that ADT may be associated with a decreased risk of second primary lung cancer. Although the protective effect of ADT against lung cancer was not as clear when the study included all of the individuals followed for more than 1 year (p=0.07), the protective effect became more statistically significant when the analysis only included patients with a follow-up period longer than 2 years (p=0.03). This strengthens the argument that ADT may have preventive effects against lung cancer development, since 2 years would be enough time for asymptomatic lung cancers to grow enough to be detected. Patients with prostate cancer typically undergo extensive surveillance imaging before starting ADT, as they are considered to have ‘high-risk’ disease. Thus, many men who were offered to start ADT for prostate cancer may have chosen not to because of newly diagnosed lung cancer, which would leave the non-ADT group with a higher number of lung cancer patients. After 2 years of follow-up, most of the initially undetected lung cancers in the non-ADT group should have grown to the point of being clinically detectable. When these cases were excluded, the difference of lung cancer incidence between the 2 groups became more significant, which provides stronger evidence for our hypothesis.

ADT with hormonal agents, such as an LHRH agonist, may modulate lung cancer risk in several different ways. Firstly, ADT may work indirectly by decreasing serum estrogen levels, thereby modulating estrogen receptors in lung tissue. Estrogen receptors have been implicated in lung cancer development in previous studies [19], and estrogen blockade was shown to be associated with decreased lung cancer risk [5]. It has been described that an LHRH agonist can suppress serum estrone and estradiol concentrations in men [20] by decreasing the amount of androstenedione and testosterone available for peripheral conversion outside of the gonads [21]. Since aromatase produced in pulmonary macrophages propagates estrogen-mediated airway inflammation [22], it is possible that a reduction in pulmonary estrogen levels from ADT may result in protective effects against second primary lung cancer.

Secondly, the decrease in androgen levels induced by ADT could directly impact pulmonary tissue. Evidence of AR expression in type II pneumocytes, bronchial epithelial cells, and lung cancer cells [7,9,23] suggests that androgens themselves may play a direct role in the development of lung cancer. Preclinical research suggests that cross-talk between ARs and the epidermal growth factor receptor may enable androgen-induced proliferation of lung cancer cells by activating mitogen activated protein kinase-dependent pathways [24]. In fact, higher baseline free testosterone levels were associated with a significantly increased risk of lung cancer development in a population-based cohort study [25].

Recently, immune modulation therapy exploiting T cell-mediated immunity, such as immune checkpoint inhibitors, have demonstrated promising results in the treatment of lung cancer [26,27]. Androgen blockage with ADT was also shown to enhance the response of AR-overexpressing prostate cancer cells to T cell-mediated killing [28]. Therefore, it is possible that ADT may exert an immunomodulatory effect on AR-expressing lung cancer cells.

Interestingly, the effect of ADT on lung cancer risk reduction was more prominent in African-American men. This could be explained by differences in sex hormonal levels; it has been shown that African-Americans have modestly but significantly higher total testosterone, estradiol, and sex hormone-binding globulin concentrations than Caucasians [29,30]. These observations of racial differences are intriguing and may be a subject for future investigation. Furthermore, the effect of ADT was more prominent in NSCLC. This may reflect the variable expression of hormonal receptors between different types of lung cancer. An *in vitro* study showed that sex steroid receptor expression was virtually absent in SCLC cell lines [7,9].

Our study utilized the VACCR as its primary data source. The VACCR has been reported to show a comparable cancer incidence to the general US men population [14]. It has been established that patients seen in the VA system tend to have a more significant smoking history [31]. As smoking is a well-described risk factor for lung cancer, the VA population is expected to have a higher proportion of people with a high risk of lung cancer, which set the stage for this study on second primary lung cancer. We calculated the standardized incidence ratio (SIR) of lung cancer in our study population and compared it to that of the general population, using SEER data. In the general US population, prostate cancer patients were less likely to develop lung cancer (SIR, 0.78) [32], but in our study population, regardless of use of ADT, prostate cancer patients tended to develop lung cancer more than expected. However, ADT users were less likely to develop second primary lung cancers than non-users (SIR, 2.59; 95% CI, 2.25 to 2.98 vs. SIR, 3.62; 95% CI, 3.27 to 4.00 (Supplementary Material 2). Furthermore, the patient population at the VA is known to include a large number of people from racial minority groups [33], which strengthens our findings of a racial disparity in the effect of ADT on second primary lung cancer.

Our study was observational, using a retrospectively constructed cohort, and was therefore prone to biases stemming from the non-random selection of treatment modality. However, smoking status, race, and follow-up between ADT users and non-users were well balanced. In addition, ADT users were older at the time of prostate cancer diagnosis, and were thus expected to have a higher risk for lung cancer, but they developed lung cancer less often than ADT non-users.

Another possible limitation of the study comes from the exclusion of a relatively large number of subjects because of missing smoking history. Because smoking is a major risk factor for lung cancer, it was considered appropriate to eliminate these subjects in order to minimize bias. The median duration of follow-up was short, which may have been related to loss to follow-up, and this could have been a source of bias. The requirement of at least 1 year of follow-up was introduced to exclude the possibility of synchronous lung cancer at the time of prostate cancer diagnosis. It also served to exclude patients who were lost to follow-up from the VA system during the first year and to ensure that all lung cancer diagnoses were captured in the VACCR. However, the exclusion of these individuals also created a risk of selection bias.

Detailed information on occupational exposure, which might have contributed to the development of lung cancer, was not available, and could have been a source of bias. However, previous carcinogen exposure on duty is not likely to affect the use of ADT after the diagnosis of prostate cancer. Owing to the very nature of cancer registry data, several specific details regarding the use of ADT were not available. We were not able to determine the modality of ADT (LHRH agonist or androgen antagonist) or the duration of use. Future investigations should include detailed data on ADT to elucidate whether modality or duration plays a role in development of second primary lung cancer.

Our study suggests that ADT may play a role in the prevention of lung cancer. The findings of our study, especially in the African-American population, provide additional support for previous studies linking steroid sex hormones to the development of lung cancer. This highlights the possibility that men may also benefit from preventive strategies targeting sex hormone conversion and signaling.

## Figures and Tables

**Table 1.** Basic characteristics of patients with at least 1 year of follow-up

Characteristics	ADT (n=7,148)	Non-ADT (n=11,559)	p-value
Lung cancer			
NSCLC	170 (88.1)	331 (87.3)	
SCLC	23 (11.9)	48 (12.7)	0.07^1^
Age (yr)^2^			
Mean (median)	70.4 (71.0)	66.4 (66.0)	<0.001^3^
Follow-up (mo)			
Mean (median)	37.0 (28.0)	36.4 (27.0)	0.88^3^
Race			
Caucasian	5,105 (71.4)	8,417 (72.8)	
African-American	1,847 (25.8)	2,784 (24.1)	
Others	196 (2.7)	458 (3.1)	0.01^1^
Smoking status			
Former	2,800 (39.2)	4,255 (36.8)	
Current	2,499 (35.0)	4,205 (36.4)	
Never	1,849 (25.9)	3,099 (26.8)	0.005^1^
Prostate cancer stage			
0	0 (0.0)	1 (0.0)	
I	31 (0.4)	76 (0.7)	
II	5,259 (73.6)	10,481 (90.7)	
III	450 (6.3)	755 (6.5)	
IV	1,408 (19.7)	246 (2.1)	<0.001^1^

Values are presented as number (%). ADT, androgen deprivation therapy; NSCLC, non-small cell lung cancer; SCLC, small cell lung cancer.

1 Chi-square test.

2 Age at the time of prostate cancer diagnosis.

3 t-test.

**Table 2.** Hazard ratios in various Cox regression models^1^

	Follow-up (yr)					
	≥1	p-value	≥2	p-value	≥3	p-value
ADT vs. non-ADT	0.85 (0.71, 1.12)	0.07	0.79 (0.64, 0.97)	0.03	0.76 (0.59, 0.97)	0.03
Age	0.99 (0.98, 1.01)	0.33	0.99 (0.98, 1.00)	0.06	0.98 (0.97, 1.00)	0.02
Caucasian vs. non-Caucasian	0.96 (0.80, 1.16)	0.70	0.96 (0.77, 1.19)	0.70	0.92 (0.71, 1.20)	0.55
Smoker vs. non-smoker	3.65 (2.72, 4.91)	<0.001	3.59 (2.54, 5.08)	<0.001	3.58 (2.38, 5.40)	<0.001
Stage 0, I, II vs. stage II, IV	0.79 (0.61, 1.03)	0.08	0.85 (0.63, 1.14)	0.29	0.98 (0.71, 1.36)	0.91

Values are presented as hazard ratio (95% confidence interval). ADT, androgen deprivation therapy.

1 Adjusted for age, smoking status, prostate cancer stage, and race.

**Table 3.** HRs of ADT for different types of lung cancer by race

Race	SCLC	p-value	NSCLC	p-value	All types	p-value
Caucasians^1^	0.98 (0.57, 1.68)	0.93	0.97 (0.82, 1.18)	0.77	0.97 (0.79, 1.19)	0.74
African-Americans^1^	0.13 (0.01, 1.11)	0.06	0.61 (0.41, 0.89)	0.01	0.56 (0.38, 0.82)	0.003
All races^2^	0.78 (0.58, 1.39)	0.36	0.86 (0.81, 1.09)	0.12	0.85 (0.81, 1.07)	0.07

Values are presented as hazard ratio (95% confidence interval). ADT, androgen deprivation therapy; SCLC, small cell lung cancer; NSCLC, non-small cell lung cancer.

1 Adjusted for age, smoking status, and prostate cancer stage.

2 Adjusted for age, smoking status, prostate cancer stage, and race.

## SUPPLEMENTARY MATERIALS

Supplementary Material 1: Table S1 is available at http://www.e-epih.org/.

Supplementary Material 2: Table S2 is available at http://www.e-epih.org/.
