# Supplementary material for: Androgen deprivation therapy is associated with decreased second primary lung cancer risk in the United States veterans with prostate cancer
Source: Epidemiol Health. 2018 Aug 11;40:e2018040. doi: 10.4178/epih.e2018040 (PMC6232654; doi:10.4178/epih.e2018040)
Supplement: Supplementary file 1 [file epih-40-e2018040-supplementary.pdf]

Supplementary Material 1

Table S1. Cohort characteristics after follow up of 2 and 3 years

	2 years			3 years		
	ADT (N=4,124)	No ADT (N=6,541)	p value	ADT (N=2,807)	No ADT (N=4,339)	p value
Lung cancer						
NSCLC	121 (89.6%)	252 (86.9%)		87 (90.6%)	185 (86.1%)	
SCLC	14 (10.4%)	38 (13.1%)	0.423¶	9 (9.4%)	30 (14%)	0.260¶
Age⌘						
Mean	70.7	67.2	<0.001§	70.7	67.5	<0.001§
Median	71	67		71	67	
Follow up(months)						
Mean	50.9	51.3	0.412§	61.6	62.3	0.142§
Median	44	44		56	57	
Race						
Caucasian	3,031 (71.9%)	4,816 (73.6%)		2,026 (72.2%)	3,226 (74.4%)	
African American	1,072 (25.4%)	1,523 (23.3%)		705 (25.1%)	991 (22.8%)	
Others	111 (2.7%)	202 (3.1%)	0.021¶	76 (2.7%)	122 (2.8%)	0.087¶
Smoking status						
Former	1,618 (38.4%)	2,368 (36.2%)		1,072 (38.2%)	1,581 (36.4%)	
Current	1,521 (36.1%)	2,484 (38%)		1,023 (36.4%)	1,688 (38.9%)	
Never	1,075 (25.5%)	1,689 (25.8%)	0.053¶	712 (25.4%)	1,070 (24.7%)	0.107¶
Prostate cancer stage						
0	0 (0.0%)	1 (0.0%)		0 (0.0%)	1 (0.0%)	
I	25 (0.6%)	49 (0.8%)		19 (0.7%)	36 (0.8%)	
II	3,146 (74.7%)	5,900 (90.2%)		2,149 (76.6%)	3,911 (90.1%)	
III	284 (6.7%)	440 (6.7%)		196 (7%)	298 (6.9%)	
IV	758(18.0%	151 (2.3%)	<0.001¶	443 (15.8%)	93 (2.1%)	<0.001¶

¶: χ^2 test §:t test

⌘: Age at the time of prostate cancer diagnosis
