# Supplementary material for: Androgen deprivation therapy is associated with decreased second primary lung cancer risk in the United States veterans with prostate cancer
Source: Epidemiol Health. 2018 Aug 11;40:e2018040. doi: 10.4178/epih.e2018040 (PMC6232654; doi:10.4178/epih.e2018040)
Supplement: Supplementary file 2 [file epih-40-e2018040-supplementary2.pdf]

Supplementary Material 2

Table S2. Standardized Incidence Ratio (SIR) of lung cancers among prostate cancer patients in VACCR and SEER database

		Observed number of lung cancer cases	Expected number of lung cancer cases	SIR	95% CI
VACCR	No ADT	379	104.98	3.62	3.27-4.00
	ADT	193	74.52	2.59	2.25-2.98
General Population (SEER)		8,135	11,621.43	0.70	$p < 0.05$
